# COVID-19 vaccine hesitancy: A narrative review of four South Asian countries

**DOI:** 10.3389/fpubh.2022.997884

**Published:** 2022-10-10

**Authors:** Farah Ennab, Rakhtan K. Qasba, Utkarsha Uday, Priya Priya, Khulud Qamar, Faisal A. Nawaz, Zarmina Islam, Nabil Zary

**Affiliations:** ^1^College of Medicine, Mohammed Bin Rashid University of Medicine and Health Sciences, Dubai, United Arab Emirates; ^2^Green Life Medical College and Hospital, Dhaka, Bangladesh; ^3^West Bengal University of Health Sciences, Kolkata, India; ^4^Faculty of Medicine, Dow Medical College, Dow University of Health Sciences, Karachi, Pakistan; ^5^Institute for Excellence in Health Professions Education, Mohammed Bin Rashid University of Medicine and Health Sciences, Dubai, United Arab Emirates

**Keywords:** COVID-19, vaccine hesitancy, public perspectives, public opinion, South Asia

## Abstract

**Objectives:**

Vaccine hesitancy remains a global issue, especially within poverty-stricken countries where there's an interplay of financial and non-financial barriers. This narrative review aims to understand attitudes and behaviors toward COVID-19 vaccination in four South Asian countries and make context-specific recommendations to vaccine program drivers and decision-makers.

**Methods:**

A search was conducted using PubMed and Science Direct, and CINHAL from January 2020 up to May 2022 restricted to the English language for terms: “Afghanistan” OR “Pakistan” OR “India” OR “Bangladesh” in combination with “COVID-19 vaccine” and other related terms. All articles were initially included, and those with relevance were included in the synthesis of this paper.

**Results:**

A narrative review was performed for this study. Our narrative review included a total of eighteen studies with a sample size (*n* = 223–5,237) averaging about 1,325 participants per study conducted. The studies included revealed public hesitancy to receive the COVID-19 vaccine ranging from 6.3 to 56.2% with an average of 31.63% across all eighteen studies. Several reasons were linked to this observation in these four South Asian countries, and the predominant ones included: Insufficient information provided to the general public about the side effects of the vaccines, concerns regarding vaccine safety, and skepticism of vaccine efficacy.

**Conclusion:**

Vaccine hesitancy is a global problem within the context of COVID-19, and issues regarding equity, misinformation, and poverty in South Asian countries makes it difficult to meet goals for herd immunity. Policymakers and governments should aim toward financial and non-financial incentives to drive the public toward vaccination.

## Introduction

Vaccines are considered to be one of the most efficacious public health intercessions in preventing further disease progression and reducing mortality rates worldwide. Over the recent years, they have been increasingly employed in various successful outbreak-related response strategies proving their essential role in the abatement of communicable diseases (1, 2). Despite the paramount evidence provided by experts in this field, there still remains a prevailing public concern globally over the safety of these therapeutic agents (3). Vaccine hesitancy, as stated by the World Health Organization (WHO) is defined as a “delay in acceptance or refusal of vaccines despite availability of vaccination services” (4). This phenomenon is being observed in many communities and especially in the South Asian region where imputable causes are plenty.

Vaccine hesitancy poses a substantial threat to tackling pandemics and most notably, the current COVID-19 pandemic which relies heavily on vaccination rings and public uptake in creating herd immunity. The causes behind this alarming phenomenon vary but can be attributed to the shaken public trust in the services provided by the healthcare systems in these countries, various doubts and mistrust of the efficacy of such interventions, and the circulated false social media claims as well as a plethora of non-factual medical statements made by religious figures in these countries (5). Additionally, socio-demographic factors have been widely studied and have shown that people residing in urban areas, those with a lower education level and a lower family economic status are more likely to be hesitant to receive the vaccine (6).

The COVID-19 situation in South Asia, in particular in Afghanistan, Pakistan, India, and Bangladesh is highly critical as these countries are among the most poverty-stricken regions of the world, accounting for a substantial portion of COVID-19 cases globally with a total count of 47,580,486 cases to date (7). By understanding the public's behaviors and attitudes toward vaccinations, we can suggest key recommendations for expanding the coverage and help correct any vaccine-related misinformation that could relate itself to the denial or active rejection of this effective tool. Furthermore, the involvement of key health policymakers in improving the containment strategies in these countries could reflect an enhanced approach to vaccination implementation. Our narrative review aims to bring national attention to an already existing problem that has been further exacerbated by the COVID-19 pandemic and to consequently use the mentioned studies' findings to help deliver context-specific recommendations to vaccine program drivers and decision-makers, thereby increasing public confidence and trust in the accessible vaccines.

## Materials and methods

**Figure 1:**
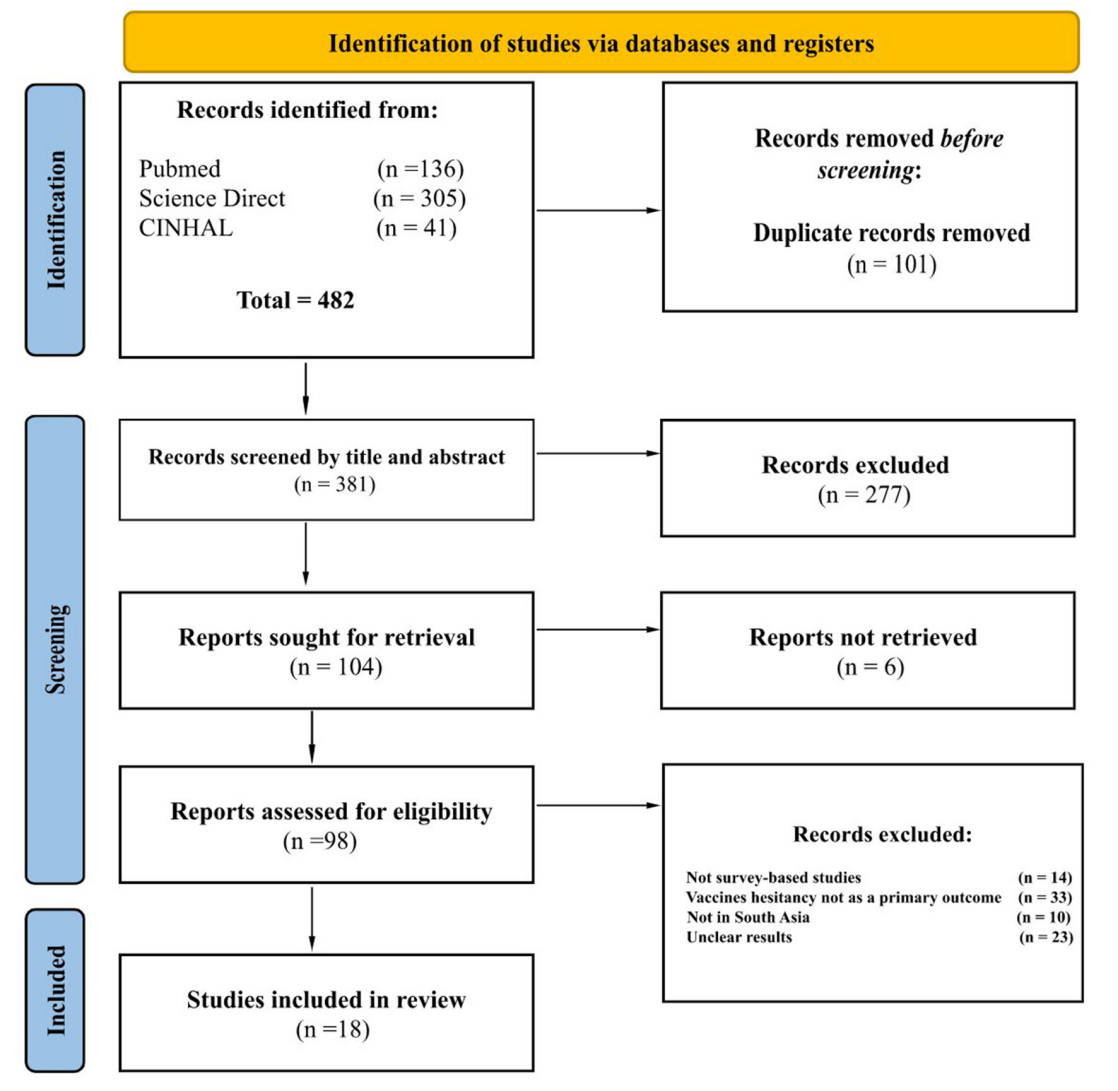
PRISMA flow diagram highlighting the selection process of the final studies included in this narrative review.

A narrative review was performed using PubMed and ScienceDirect, and CINHAL from January 2020 up to May 2022. The search was restricted to the English language, in order to identify COVID-19 vaccine hesitancy in the included countries. The last search was performed on the 9th of May 2022. We included the following search terms: “Afghanistan” OR “Pakistan” OR “India” OR “Bangladesh” in combination with “COVID-19 vaccine” and “vaccine hesitancy” with any other relevant and identified synonyms. We included only survey-based studies with a primary outcome of COVID-19 vaccine hesitancy, which were conducted in South Asian countries. These studies investigated the perception, vaccine confidence, and vaccine hesitancy in these populations Following this initial literature review, we only included the articles which were pertinent to our research aims. The final database of studies included one of the four aforementioned countries with a clear focus on COVID-19 vaccine hesitancy amongst the public (Figure 1).

## Results

The final pool of the studies comprised a sample size ranging from 223 to 5,237 participants with an average study sample of 1,325 and a total of 23,854 participants across all eighteen studies included (8–25). Our review included a total of one study from Afghanistan, five from Bangladesh, eight from India and four from Pakistan. Amongst the studies that explored reasons for vaccine hesitancy, it was noted that insufficient information provided to the public and increased concerns about vaccine safety and efficacy were identified as being the major arguments for COVID-19 vaccine refusal and hesitancy. Additionally, other reported reasons included the public perception that the COVID-19 vaccines might low-quality. Another common argument made was that people did not anticipate being infected with the virus hence, they did not feel the urge to seek the vaccines. From these four South Asian countries, we concluded that the prevalence of vaccine hesitancy for COVID-19 varied from 6.3 to 56.2% with an average rate of 31.63%.

Study participants included in this narrative literature review differed in age, gender, ethnicity, profession, the highest level of education attained, financial income, and marital status (Table 1).

**Table 1:** Survey-based cross-sectional studies included in this narrative review relevant to COVID-19 vaccine hesitancy factors in Afghanistan, Pakistan, India, and Bangladesh.

**SL**	**References**	**Country of origin**	**Study design**	**Time period**	**Study population**	**Sample size (*n*)**	**Prevalence of vaccine hesitancy (%)**	**Factors related to COVID-19 vaccine hesitancy**
1	Nemat et al. (8)	Afghanistan	Cross-sectional	December 2020–January 2021	General population	806	42.4%	• “Lower income countries are supplied with low-quality vaccines” • “Vaccines are unsafe” • “I have enough immunity” • “Vaccines will be expensive”
2	Patwary et al. (9)	Bangladesh	Cross-sectional	5th of July 2021–August 2021	Bangladeshi residents over the age of 18	543	15%	• “Unknown side effects causing fear” • “Vaccines are not effective enough” • “Not enough information is provided on vaccines” • “Financial burden of the vaccines” • “COVID-19 is harmless” • “Natural immunity is better than vaccination” • “I have contraindications to the vaccines” • “I prefer if other people get vaccinated first”
3	Alam et al. (10)	Bangladesh	Cross-sectional	3rd of January 2021–25th of January 2021	Healthcare workers	831	33%	• “Unknown side effects of vaccines” • “ Vaccine quality may be compromised due to mass production in a rush”
4	Hossain et al. (11)	Bangladesh	Cross-sectional	22nd of March 2021–1st of April 2021	Public university students	900	56.2%	• “I am worried about the vaccine side effects” • “I am not worried about COVID-19” • “Pandemics can be reversed without vaccines” • “Antibiotics can heal COVID-19 disease” • “Vaccines can be only applied to people who have been infected with COVID-19” • “Eating immune system boosting food can outperform vaccines” • “Vaccines should be given to patients with chronic health conditions”
5	Ali et al. (12)	Bangladesh	Cross-sectional	10th of October 2021–31st of October 2021	Parents aged ≥ 18 years and having at least one child aged <18 years diagnosed with neurodevelopmental disorders	396	42.7%	• “Vaccines are not safe and effective for Bangladeshi children” • “None of my family members tested positive for COVID-19” • “None of my family members died of COVID-19” • “COVID-19 cannot infect us” • “We are not concerned at all about our children getting infected”
6	Ali et al. (13)	Bangladesh	Cross-sectional	10th of October 2021–31st of October 2021	Parents aged ≥ 18 years with children <18 years of age	2,633	42.8%	• “Vaccines are not safe and effective for Bangladeshi children” • “I didn't receive a vaccine as a parent” • “None of my family members could be infected with COVID-19” • “We are not concerned at all about our children getting infected”
7	Kumar et al. (14)	India	Cross-sectional	During 2021	General population	841	27.2%	• “Concerns about vaccine safety” • “Antivaccine attitude and beliefs” • “Concerns of fear and phobia” • “New vaccine” • “Not in risk group” • “Lack of information”
8	Jain et al. (15)	India	Cross-sectional	2nd of February 2021–March 2021	Medical students	1,068	10.6%	• “Mistrust in vaccine safety” • “Vaccines are not efficacious enough” • “Young age” • “No need for vaccines as COVID-19 pandemic is over now” • “Previous COVID-19 exposure”
9	Joshi et al. (16)	India	Cross-sectional	10th of April 2021–10th of June 2021	Healthcare workers	223	6.3%	• “Insufficient information regarding the vaccine” • “Fear of unknown adverse effects” • “Doubt in vaccine effectiveness” • “Distrust in vaccine companies” • “Fear of vaccine's side effect on current pregnancy”
10	Jacob et al. (17)	India	Cross-sectional	2nd of January 2021–14th of January 2021	All adults over the age of 18	2,032	21.4%	• “Unknown side effects of the available vaccines” • “Vaccines are unnecessary” • “Mistrust in the country's authority” • “No perceived risk of COVID-19 infection” • “Cost of vaccine is not affordable”
11	Mathur et al. (18)	India	Cross-sectional	January 2021–February 2021	Healthcare workers	3,102	33.6%	• “I am worried about the side effects of the vaccine” • “Fear of needle prick” • “Fear of vaccine-induced COVID-19-like illness” • “Vaccine may be ineffective”
12	Singh et al. (19)	India	Cross-sectional	January 2021	Healthcare workers	254	35.8%	• “Not sure about the efficiency of vaccine” • “Worried about the side effects” • “Worried about effects of the vaccine on mental health” • “I am already infected; no need to vaccinate now”
13	Danabal et al. (20)	India	Cross-sectional	During 2021	All adults over the age of 18	564	40.7%	• “ COVID 19 is not real” • “Vaccines are not powerful enough for this new virus COVID-19” • “Vaccines cause serious problems in children” • “Unknown long-term side effects of vaccine” • “COVID-19 vaccination is politically motivated” • “Vaccination programs are deceitful” • “Natural immunity lasts longer than vaccines” • “Natural exposure gives more protection”
14	Ekstrand et al. (21)	India	Cross-sectional	18th of January 2021–19th of February 2021	Individuals aged ≥18 years and diagnosed with HIV	438	40%	• “Lack of confidence in vaccines” • “Concerned about side effects” • “Distrust in vaccines”
15	Malik et al. (22)	Pakistan	Cross-sectional	3rd of December 2020 −14th of February 2021	Healthcare workers	5,237	24.5%	• “I have some religious concerns” • “Vaccines are not effective enough” • “Fear of vaccine side effects” • “Chronic comorbidities such as allergies, etc..” • “Previous exposure to COVID-19 infection”
16	Tahir et al. (23)	Pakistan	Cross-sectional	27th of September 2020–11th of October 2020	All adults over the age of 18	883	29.2%	• “COVID-19 is not a serious disease • “COVID-19 is a conspiracy” • “Vaccines have no role in disease prevention” • “I would become infected due to the vaccination” • “Unknown side effects are worrying me” • “Natural immunity is better than the vaccination” • “I am using protective measures against COVID-19” • “I am afraid of needles” • “I cannot afford the vaccine” • “I am concerned if the vaccine is “halal”” • “Vaccines are not properly stored in our country”
17	Yasmin et al. (24)	Pakistan	Cross-sectional	28th of January 2021–11th of February 2021	All adults over the age of 18	1,778	28%	• “I am concerned about side effects” • “I don't need a vaccine as I follow all preventive measures seriously” • “I don't believe the vaccine will stop the infection” • “COVID-19 vaccination is a conspiracy” • “I am young, healthy, and immune” • “I am afraid of needles”
18	Zak et al. (25)	Pakistan	Cross-sectional	July 2021–September 2021	Individuals aged ≥ 40 years	1,325	40%	• “Vaccines have side effects and are unsafe” • “It is not useful” • “Vaccine is not effective enough” • “My immune system is strong enough” • “There is no COVID” • “Vaccination is a Western/Jews/Israeli/American/Illuminati plot” • “I am stressed out” • “Religious reason” • “Due to some chronic conditions” • “Social pressure” • “Covid-19 vaccine-related stress/anxiety” • “Prior Covid exposure leads to the development of antibodies”

## Discussion

Vaccine hesitancy has been a prevailing concern reported by policymakers at varying levels, across as many as 90% of countries worldwide (26). Since the outbreak of the pandemic in 2020, a long journey of at least partially vaccinating 64.5% of the population across the globe, has been traversed (27). The issue of vaccine hesitancy remains a growing phenomenon, particularly during the COVID-19 pandemic. Unduly vaccine development efforts resulting in poor vaccine efficacy, and adverse reactions have been reported as some of the reasons behind the public's refusal to seek vaccines. Usually, the acceptability of a vaccine is said to have been influenced by the level of awareness about a disease, availability, and accessibility to a healthcare commodity (28).

Approximately, 49% of Pakistani citizens were reluctant to receive the COVID-19 vaccine (29). As of the 30th of April 2022, around 59.65% of the residents had been vaccinated (27). A literature review by Nemat et al. reported that about 88% of Afghanis were aware of the efforts being made to develop vaccines for COVID-19. They also observed a significantly higher number of females than males, eager to receive the COVID-19 vaccine, this comes in contrast to a European survey suggesting the opposite (8). Paterson et al. reported that vaccine hesitancy is also prevalent among healthcare workers and especially medical students (30, 31). Since January 2021, the COVID-19 vaccination programs in India had initially prioritized the frontline healthcare workers and then gradually, spread its programs to cover the rest of the population. It is noteworthy to mention that 61.98% of India's citizens are fully vaccinated (27). Abedin et al. observed that 74.5% of Bangladeshi citizens were keen to get the COVID-19 vaccination, resonating with France, Australia, Mexico, India, and Ireland, as confirmed by a population-based study conducted in these countries (32).

In addition, the assumed poor vaccine quality amongst the public, growing concerns over vaccine safety, and efficacy, rumors about clots during menstruation, and infertility have led to apprehensions regarding the COVID-19 vaccine. Few people even believed that the available vaccines may increase the mortality rate (6). Trust in the government due to inaccessible and inequitable distribution of economy and healthcare facilities among the communities, is also one of the major factors contributing to vaccine hesitancy. Furthermore, lack of technological literacy and poor refrigeration facilities adds to the dissimilar distribution of the COVID-19 vaccines across different regions in the same country.

Executing an effective mass vaccination drive demands the addressal of COVID-19 vaccine hesitancy. It is also essential to acknowledge various other factors which play an important role in these countries such as societal beliefs and literacy rates. Mass vaccination should be aimed at addressing the factors leading to vaccine hesitancy *via* interventions tailored to societal concerns and parameters, not restricted to any specific region.

The aim of the review is to illustrate the prevalence and describe the predictors of the COVID-19 vaccine hesitancy, in Pakistan, Afghanistan, India, and Bangladesh, with the latest available evidence, thereby, increasing the literature coverage in scoping. This will invaluably aid the various programs promoting vaccinations to raise awareness while addressing individual, economic, socio-cultural, political, and regional barriers. Specific proposals and recommendations formulated with the aid of public-private partnerships (PPP) would go a long way in combating this problem. The key to success in attaining herd immunity against COVID-19 mostly relies on the public uptake of the vaccines available. However, new emerging viral mutants, formed due to rapid antigenic shift and drift, are a constant challenge, which demands the attention of researchers worldwide.

### Current efforts to combat COVID-19 vaccine hesitancy in these countries

In Pakistan, various efforts are being made at different levels to raise awareness about the efficacy of the COVID-19 vaccine, these include radio messages and large-scale video transmissions on TV and the internet providing the necessary adequate knowledge about vaccines and empowering the general public to accept the COVID-19 vaccine, as well as door-to-door vaccine administration and awareness drives similar to those used for Polio (33). Meanwhile, the government in India is not making any significant efforts to combat vaccine hesitancy; nevertheless, a “time-bound inquiry” into deaths that occurred soon after vaccination was ordered, and each mobile phone call in the country was automatically initiated by a national programmed message from the Indian government affirming the safety and effectiveness of vaccines (34). Several initiatives are being implemented in Afghanistan to prevent vaccination hesitancy, including routine immunization vaccinators and the deployment of 2,000 more new health professionals (teams of two people: one male and one female), raising societal awareness and educating them, and avoiding myths (35). Similarly, in Bangladesh, the most popular variables contributing to decreasing vaccination hesitancy include social media and awareness campaigns (36).

To lessen the impact of vaccine hesitancy, it is imperative to critically analyze the situation based on different countries. Each country has a unique context that should be taken into consideration. Hence, varied policies are need to be enacted so as to ensure that each country can impede the impact of vaccine hesitancy.

### Effects and recommendations of COVID-19 vaccine hesitancy in Afghanistan

In Afghanistan, the presence of conflict, illiteracy, and poverty has favored the condition for COVID-19 to continue spreading. Despite the country's high trends of other problems, the virus has only become a normal disease for some people. The continued spread has also impacted the uptake of the vaccines. A study conducted in the capital of Afghanistan, Kabul, revealed that 37% of the population is hesitant to receive the vaccine. In order to improve the situation, community engagements to raise awareness about the harmful effects of COVID-19 and the positive effects of the vaccines are important to be conducted. Moreover, social media awareness is also considered essential to improve the public's perception. However, it must be monitored to detect any source of misinformation and immediately stifle it. Lastly, in a country like Afghanistan, people pay great attention to religion and religious figures. Therefore, religious figures' engagement is crucial to raise awareness about important aspects of the vaccines (8).

### Effects and recommendations of COVID-19 vaccine hesitancy in India

The Republic of India is not strange in facing vaccine hesitancy. This longstanding problem has resulted in a deferment in achieving the vaccination target for COVID-19. However, the Indian government is firmly strengthening its vaccination drives, *via* mass, print as well as social media coverage to help burst the myths surrounding the COVID-19 vaccines. It is also imperative to encourage joint efforts between district-level administrations and political leaders to dispel the hoax around COVID-19 vaccines through awareness sessions using regional folk songs.

Nevertheless, it is critical for the nation to develop an effectively sustainable campaign to tackle vaccine hesitancy. The government should invest in evidence-based research, as a public-private partnership, identify the population strata with distrust in vaccines with a resolute to resolve their hesitancy to expand wide immunization coverage. A versatile team comprising of experts from different fields such as immunology, pharmacology, microbiology, behavioral science, and sociology should be formulated at the national as well as, regional levels to conduct rigorous research and come up with solutions to help people accept the COVID-19 vaccines.

Communicating the advantages of vaccines in colloquial languages, backed by strong methodological proof of vaccine safety and efficacy, in addition to street plays to raise awareness, would pave the way in building the confidence of the masses in vaccines. Optimizing the support of mass media communications, and public posters to dismiss the hearsay and promote vaccines, besides, door-to-door campaigns conducted by social healthcare workers might be pivotal in instilling trust in vaccines (37).

Furthermore, rapid interventions are needed to accelerate the COVID-19 vaccination availability across the healthcare sectors and especially among individuals seeking the vaccines but facing inaccessibility to the vaccination centers. In order to encourage mass coverage, the administration should either make the vaccination available free of cost or provide reimbursement of the charges or tie up with the health insurance companies to cover the cost. Non-financial incentives, such as complimentary food items or a free health check-up, may also help out in the intention of combating vaccine hesitancy (38). Such sustained financial or non-financial incentives for vaccination coupled with public engagements would gauge the doubtfulness of the public and addresses their growing concerns.

### Effects and recommendations of COVD-19 vaccine hesitancy in Bangladesh

According to many surveys conducted in Bangladesh, there has been significant vaccine hesitancy shown by the general public owing to personal beliefs, mistrust, religious factors, conspiracy theories, and concerns about vaccine safety - all of which have contributed to widespread misconceptions regarding vaccines. These incidents demand the immediate attention of Bangladesh's public health officials (9).

To clarify unfavorable public perceptions against the vaccination, an effective communication campaign engaging community members should be planned and conducted. Furthermore, it is paramount to ensure that accurate information on the COVID-19 vaccine procedure is constantly disseminated *via* effective media channels, such as the internet, TV news, and social media websites (39). Through these outlets, public health messages emphasizing faith in vaccination safety, efficacy, and benefits can be quite helpful. Public officials and national figures who have received the COVID-19 vaccination might also share their experiences in the media to urge others to become immunized. The authority should expand the number of community-based clinics and vaccination booths for online registration and immunization. With adequate administration, walk-in vaccination programs might be addressed. They can add extra personnel to properly handle the entire process. To combat this deadly disease, authorities must equip and teach their staff and other essential players. Furthermore, extensive coordination among academics, authorities, and societies is required to design a successful COVID-19 immunization program for all individuals (40).

All of these measures should be used by the authority to carry out its policy of broad COVID-19 immunization coverage. While it is challenging to manage misconceptions, it is essential to recognize inaccurate medical statements and circulated myths and work on rather promoting sound scientific facts regarding vaccination.

### Effects and recommendations of vaccine hesitancy in Pakistan

Pakistan is also dealing with the rising issue of COVID-19 vaccination reluctance. It is one of the countries with the lowest vaccination rates. During these threatening times, COVID-19 vaccine hesitancy remains a significant barrier to Pakistan's public health. People from lower socioeconomic levels are less likely to be vaccinated. Fear of the vaccine's safety and efficacy, potential ill effects, lack of faith in vaccine-development institutions, and concern that the vaccination might cause autism, infertility, autoimmune diseases, and death are all factors impacting public adoption of the COVID-19 vaccine. As a result, Pakistan urgently needs to establish a stronger healthcare system to curb viral transmission (33, 41).

To urge people to get vaccinated against COVID-19, the Pakistani media must refrain from broadcasting anything that fuels conspiracy theories about the virus. An online telehealth programs should be established so that the any member of the public may direct their queries and concerns to specialists, who can reply and comment on vaccination safety. In the country, mass awareness campaigns should be conducted using various social media apps, TV channels, radio programs and newspapers as well. The priority should be placed on the importance of immunization by noting prior vaccine achievements (41). Consideration should be given to the provision of financial incentives for vaccination. Religious conspiracies and erroneous beliefs about vaccines containing pig or monkey derivatives should be reduced by incorporating the religious experts and have them educate the general public about the necessity of immunization in accordance with Islamic Sharia law (23).

The major cause of vaccine refusal in this country is the lack of scientific understanding about vaccination among the general people; hence, the WHO must step up its duties to effectively address public inquiries and provide the most up-to-date scientific information about the available vaccines.

## Limitations

This paper has several limitations. Given the nature of narrative reviews, the articles included in our study were not systematically reviewed, hence there exists an area for selection bias. Moreover, articles in the English language were only included which may have prevented us from accessing literature in other native languages across South Asia. In addition, conference proceedings and other databases such as Scopus were not included in our search which limited our final results. Our review only included cross-sectional studies that were survey-based while other studies including ones that analyze threads on social media platforms may have provided more insights since the use of such platforms increased during the pandemic. Also, qualitative studies may have given more in-depth descriptions of individual experiences. Collectively, these different factors could potentially add bias, and varying views may reflect different findings suggesting the diversity of opinions and conclusions.

## Conclusion

With the rise in COVID-19 cases amidst new variants on a global scale, there is a strong need to tackle socio-economic challenges to vaccine uptake in developing countries, including vaccine hesitancy in the general population. Lower-and-middle income countries such as Afghanistan, Bangladesh, India, and Pakistan have shown the unique challenges to vaccinations along with lessons on successful implementation of cost-effective strategies in these regions. Further research is warranted on the role of vaccine misinformation and recommendations for unified health governance on this crucial matter.

## Author contributions

FE contributed to the conceptualization of this manuscript, writing of the original draft, and review and editing. RQ contributed to the methodology, analysis of the data, and literature review. UU, PP, KQ, FN, and ZI contributed to the writing of the original draft. NZ contributed to the review and editing of the original draft. All authors contributed to the article and approved the submitted version.

## Funding

This study received funding in part from Al Jalila Foundation in addition to a Pfizer Independent Medical Education Grant (Agreement No. 67504787). The funders were not involved in the study design, collection, analysis, interpretation of data, the writing of this article or the decision to submit it for publication.

## Conflict of interest

The authors declare that the research was conducted in the absence of any commercial or financial relationships that could be construed as a potential conflict of interest.

## Publisher's note

All claims expressed in this article are solely those of the authors and do not necessarily represent those of their affiliated organizations, or those of the publisher, the editors and the reviewers. Any product that may be evaluated in this article, or claim that may be made by its manufacturer, is not guaranteed or endorsed by the publisher.
